# Long-Term Weight Loss Results, Remission of Comorbidities and Nutritional Deficiencies of Sleeve Gastrectomy (SG), Roux-En-Y Gastric Bypass (RYGB) and One-Anastomosis Gastric Bypass (OAGB) on Type 2 Diabetic (T2D) Patients

**DOI:** 10.3390/ijerph17207644

**Published:** 2020-10-20

**Authors:** Maria-Jose Castro, Jose-Maria Jimenez, Miguel-Angel Carbajo, Maria Lopez, Maria-Jose Cao, Sara Garcia, Jaime Ruiz-Tovar

**Affiliations:** 1 Nursing Faculty, University of Valladolid, 47002 Valladolid, Spain; mariajose.castro@uva.es (M.-J.C.); maria.lopez.vallecillo@uva.es (M.L.); mjcao@enf.uva.es (M.-J.C.); sara.garcia.villanueva@uva.es (S.G.); 2 Centre of Excellence for the Study and Treatment of Obesity and Diabetes, 47004 Valladolid, Spain; doctormacarbajo@gmail.com (M.-A.C.); jruiztovar@gmail.com (J.R.-T.); 3 Department of Surgery, Rey Juan Carlos University, 28933 Madrid, Spain

**Keywords:** obesity, type 2 diabetes, one-anastomosis gastric bypass, Roux-en-Y gastric bypass, sleeve gastrectomy

## Abstract

This study aimed to compare the long-term weight loss results, remission of comorbidities and nutritional deficiencies of sleeve gastrectomy (SG), Roux-en-Y gastric bypass (RYGB) and One-Anastomosis gastric bypass (OAGB) on type 2 diabetic (T2D) patients. Patients and Methods: A retrospective analysis of all the morbidly obese and diabetic patients undergoing SG, RYGB, and OAGB as primary bariatric procedures between February 2010 and June 2015 was performed. Anthropometric parameters, remission of comorbidities, nutritional deficiencies and supplementation requirements at 1, 2 and 5 years’ follow-up were monitored. Patients lost to follow-up 5 years after surgery were excluded from the analysis. Results: 358 patients were included. The follow-up rate was 84.8%. Finally, 83 SG, 152 RYGB, and 123 OAGB patients were included in the analysis. OAGB obtained significantly greater weight loss and remission of dyslipidemia than the other techniques. There was a trend towards greater T2D and hypertension remission rate after OAGB, while fasting glucose and glycated hemoglobin levels were significantly lower after OAGB. There were no significant differences in hemoglobin or protein levels between groups. SG obtained lower iron deficiencies than the other techniques, while there were no significant differences in other nutritional deficiencies between groups. Conclusion: OAGB obtained greater weight loss and remission of dyslipidemia than RYGB or SG. Excluding lower iron deficiency rates after SG, there were no significant differences in the development of nutritional deficiencies between groups.

## 1. Introduction

Obesity and its comorbidities are a global health problem, mainly in developed countries. In the last decades, morbid obesity has shown an exponential growth [1]. Bariatric surgery has demonstrated to be the most effective method in achieving sustained weight loss and reducing obesity-related comorbidities, especially type 2 diabetes mellitus (T2D) and cardiovascular risk [2,3,4]. Thus, the number of bariatric procedures is continuously increasing, and surgical concepts are evolving in pursuit of an "ideal technique” that would address long term weight loss and remission of comorbidities with minimal nutritional burden [5].

Roux-en-Y gastric bypass (RYGB) and sleeve gastrectomy (SG) are the most frequently performed bariatric procedures world-wide [6]. However, One-Anastomosis Gastric Bypass (OAGB) is an increasingly performed procedure, as it is associated with low complications and excellent short- and long-term benefits regarding weight loss and remission of comorbidities [7]. Few comparative studies are available, and the results are controversial. Our group has recently published a prospective randomized trial comparing OAGB, RYGB, and SG, observing greatest weight loss and remission of comorbidities in the OAGB group, whereas nutritional deficiencies were comparable between RYGB and OAGB and significantly lower after SG [8]. However, the YOMEGA trial, comparing OAGB with RYGB, determined no significant differences in weight loss between groups after 2 years of follow-up, but significantly greater serious adverse events, mostly nutritional complications, after OAGB [9]. The SLEEVEPASS trial compared the long-term results obtained after SG and RYGB, observing a greater weight loss after RYGB, but without significant differences in the remission of comorbidities [10].

Despite initial medical treatment of T2D patients consisting of a caloric restriction and including weight loss as a target for improving the glycemic control, after bariatric surgery, the improvement in glucose homeostasis typically begins within days of surgery before significant weight loss occurs. Thus, total body weight loss per se is unlikely to play a significant role in mediating early glycemic improvements. The physiological and molecular mechanisms underlying the beneficial glycemic effects of bariatric surgery remain incompletely understood. Diverse mechanism have been proposed to contribute to the glycemic control: proximal intestinal nutrient exclusion, rapid distal gut nutrient delivery, changes in bile acid metabolism, gastrointestinal tract nutrient sensing and glucose utilization, incretins, possible anti-incretin(s), or the intestinal microbiome. These changes, acting through peripheral and/or central pathways, lead to reduced hepatic glucose production, increased tissue glucose uptake, improved insulin sensitivity, and enhanced b-cell function [11].

This study aimed to compare the long-term weight loss results, remission of comorbidities and nutritional deficiencies of these three bariatric techniques on a selected population of T2D patients.

## 2. Materials and Methods

### 2.1. Study Design

A retrospective analysis of all the morbidly obese and diabetic patients undergoing SG, RYGB, and OAGB as primary bariatric procedures between February 2010 and June 2015 was performed. 

Inclusion criteria were body mass index (BMI) >40 Kg/m^2^ or BMI > 35 Kg/m^2^ with the presence of comorbidities associated with obesity and age older than 18 years. Exclusion criteria were patients undergoing bariatric techniques other than SG, RYGB and OAGB, and patients undergoing any other surgical procedure added to the bariatric surgery.

### 2.2. Preoperative Evaluation

A multidisciplinary team, including surgeons, endocrinologists, dieticians, and psychologists, performed a work-up to evaluate potential surgical candidates. A dietician established a diet consisting of total daily energy intake of 1200 Kcal. Given the prolonged waiting list for bariatric surgery at our institutions, a weight loss of at least 10% of the patient’s weight was considered an indispensable condition to undergo the surgery. In this way, the patients who could benefit most from surgery were selected according to the results of previous studies of our group [12,13,14].

Psychologists assessed additional interviews to evaluate the implication of the patient with the consequences of the surgery in order to guarantee an adequate follow-up scheme and determine if the selected patients would have total adherence to the recommended diet and supplementation. In our preoperative evaluation protocol, this thorough selection was applied to all the candidates for bariatric surgery.

Patients received information from the bariatric surgeon, the endocrinologist and the dietician about possible perioperative complications, eventual postoperative deficiencies and necessary nutritional supplementation.

### 2.3. Surgical Techniques

All procedures were performed laparoscopically, described in the literature previously [7,8]. Access was made through 5 ports in the SG and RYGB techniques, unlike OAGB with 6 ports. In all cases, a linear stapler (Endo-GIA with three-staple cartridges, Covidien, USA) was used to suture the gastric sac.

In the 3 techniques described, after surgery, the recommendations at hospital discharge were as follows: advice from the medical team, nutritional support and specific exercise adapted to each patient. In addition, mineral and multivitamin supplementation (Elevit^®^, Bayer^®^, Leverkusen, Germany) was prescribed in all three groups (2 tablets/day).

### 2.4. Follow-Up

All the patients were followed up by the surgeon and endocrinologist every 3 months during the first postoperative year, every 6 months during the second year after surgery and then annually throughout life. Anthropometric parameters and comorbidities resolution were evaluated. Blood samples were obtained during all the follow-up visits to evaluate possible nutritional deficiencies and prescribe necessary supplementation according to laboratory results. Once a nutritional deficiency was established, specific supplementation was prescribed. When serum values of this deficiency were normalized in posterior controls, the specific supplementation was stopped.

Medical treatment, such as antidiabetic, antihypertensive and hypolipemiant drugs, was adjusted according to the current needs of the patient.

### 2.5. Remission of Comorbidities

Remission of associated comorbidities was defined in the case of type 2 diabetes mellitus (T2D): plasma glucose <100 mg/dL and glycated hemoglobin (HbA1c) below 6% in the absence of hypoglycemic treatment. The variables to determine the remission of arterial hypertension (HT) were <135 mmHg in systolic blood pressure and <85 mmHg in diastolic pressure in the absence of antihypertensive treatment. Among the lipid profile variables, a remission of dyslipidemia (DL) was considered if total cholesterol <200 mg/dL, triglycerides <200 mg/dL, and high-density lipoprotein cholesterol>40 mg/dL in the absence of pharmacological therapy.

### 2.6. Variables

Anthropometric variables were analyzed: weight, BMI and excess BMI loss (EBMIL). The assessment of the remission of the studied comorbidities (T2DM, HT, and DL), the specific needs for vitamin and mineral supplementation, hemoglobin, albumin, total proteins, lipid and glycemic profile were determined before surgery (baseline), 1, 2 and 5 years after surgery. A retrospective database was performed including all these variables.

### 2.7. Statistical Analysis

Data were analyzed using IBM SPSS v. 22.0 software (IBM, Armonk, New York, NY, USA). Quantitative variables that followed a normal distribution were summarized as means ± standard deviations (SD). Medians and ranges were recorded for non-Gaussian variables. Qualitative variables were summarized by number and as percentage of cases. A comparison of quantitative variables between the 3 groups was conducted using analysis of variance (ANOVA). A comparison of qualitative variables was performed with the chi-square test; in cases with fewer than 5 observations in the cell, the Fisher exact probability method was used. Pairwise comparisons of groups used a Bonferroni corrected *p*-value. Significance was set at *p*-value < 0.05.

### 2.8. Ethics Statement

This study was approved by the University of Valladolid Institutional Review Board and the Ethics Committee of the Faculty of Nursing (Identification code: 2020/JMJP01). Informed written consent was obtained from all subjects before undergoing the bariatric procedure and includes their acceptance of using their personal data anonymously with research purposes. The study adhered to the Declaration of Helsinki (2013). 

## 3. Results

A total of 358 T2D patients were included for analysis in the present study, 273 women and 85 males, with a mean age of 43.3 ± 10.9 years and a mean BMI of 44 ± 9.1 Kg/m^2^. All the patients were under oral antidiabetic treatment and 23.2% under insulin therapy. Baseline features and distribution of patients by the procedure are summarized in Table 1. There were no significant differences in the baseline features of the patients between groups. However, it is remarkable that the prevalence of dyslipidemia and hypertension is greater among this T2D population than that reported in other studies, including T2D and non-T2D patients.

Postoperative complications included two staple line leaks after SG, one gastro-jejunal anastomotic leak and one hemoperitoneum after RYGB, and one anastomotic leak and two cases of intraperitoneal bleedings after OAGB.

The patients lost to follow-up at 5 years postoperatively were excluded from the analysis of weight loss, remission of comorbidities, laboratory data, and needs for supplements, even if there were data available at 1 and 2 years’ follow-up. From all the patients operated, the follow-up rate at 5 years postoperatively was 87.2% in the SG group, 83.2% in RYGB and 85.7% in OAGB. Finally, 83 patients were analyzed in group SG, 152 in RYGB and 123 in OAGB.

### 3.1. Postoperative Anthropometric Measurements

Postoperative BMI and EBMIL are summarized in Table 2. The pairwise analysis revealed that BMI after OAGB was significantly lower than after RYGB and SG at 1, 2 and 5 years after surgery (*p* < 0.001, in all the determinations), while there were no significant differences between RYGB and SG at any of the time points. Similarly, EBMIL after OAGB was significantly higher than after RYGB and SG at all the determinations (*p* < 0.001, respectively), but without reaching statistical significance between RYGB and SG. There were no cases of BMI below 19 Kg/m^2^ in any of the groups.

### 3.2. Remission of Comorbidities

The remissions of comorbidities are shown in Table 3. Remissions of T2D and DL were significantly greater after OAGB but HT did not achieve statistically significant differences between groups, despite the fact that there is a trend towards higher remission rates after OAGB. Referring to T2D, a significant association between duration of T2D and complete remission could not be observed at 1, 2 and 5 years after surgery. However, when stratifying the sample according to the preoperative treatment with insulin, a complete remission of T2D could be observed in 27.7% after SG, 38.8% after RYGB and 69% after OAGB at 1 and 2 years after surgery (*p* = 0.010). At 5 years postoperatively, the complete remission rate was observed in 16.7% after SG, 30.8% after RYGB and 66.7% after OAGB (*p* = 0.0005).

When stratifying the sample, according to the preoperative treatment for T2D, with or without insulin, significantly lower remission rates were determined for patients under preoperative insulin therapy and undergoing RYGB or OAGB. For patients undergoing SG, there was a trend towards lower remission rates. Thus, it can be observed that preoperative insulin needs are a risk factor for lower remission rates (Table 4).

Analyzing exclusively the patients under insulin treatment, significant differences in complete remission rates of T2D could not be determined between the different techniques (Table 5).

The duration of T2D was not associated with the complete remission rates of T2D in any group.

### 3.3. Laboratory Data

Referring to the glycemic and lipid profile, OAGB obtained significantly lower fasting glucose, HbA1c, total cholesterol, and triglycerides levels than the other two techniques at 1, 2 and 5 years after surgery, while HDL-cholesterol values were significantly lower after RYGB when compared with SG and OAGB. Pairwise analyses, comparing OAGB with RYGB, revealed significantly lower levels of glucose, glycated hemoglobin, total cholesterol and triglycerides after OAGB at the three time points (Table 6).

There were no significant differences in hemoglobin, albumin and total protein levels between groups (Table 7). Referring to nutritional deficiencies, only iron deficiencies were significantly lower after SG at 5 years’ follow-up. The rest of the nutritional deficiencies were comparable between groups during the whole follow-up time points.

## 4. Discussion

In the present study, we compare 358 T2D patients undergoing OAGB, RYGB, and SG, aiming to evaluate long-term weight loss, remission of comorbidities and nutritional deficiencies. The two main strengths of the present paper are the long-term results and the high follow-up rate at our institutions. We describe a follow-up of over 80% of the patients in the three groups. The recently published YOMEGA trial [9], comparing RYGB and OAGB at 2 years’ follow-up, reports that over 20% of patients are lost to follow-up. We should consider that patients undergoing any type of bariatric approach are at risk of developing nutritional deficiencies and must be closely and lifelong followed. Higa et al. [15] reported that over 80% of patients undergoing RYGB have some type of nutritional deficiency. We assume that a lifelong follow-up is an enormous healthcare burden. Therefore, multidisciplinary teams are necessary also for the postoperative course, including endocrinologists, dietitians, and general practitioners, but all of them must be in permanent contact to assure correct postoperative management of the patient. Consequently, if a close follow-up cannot be assured because of logistic problems or lack of implication of the patient, the indication for any bariatric technique should be reconsidered, as the potential nutritional sequelae can be serious.

In the present study, a significantly greater weight loss in the OAGB group could be observed at 1, 2 and 5 years. Five years after surgery, the mean BMI in the OAGB group was 25.9 Kg/m^2^, whereas in the RYGB and SG groups, the mean BMI was in the range of obesity, and the weight curve shows a clear weight regain 5 years after surgery. At a 2 years’ follow-up, the weight loss that we obtained in the OAGB group is higher than that reported in the YOMEGA trial [9]. Lee et al. [16] also conducted a randomized clinical trial comparing RYGB (with 100 cm of biliopancreatic limb and 150 cm of alimentary limb) with OAGB (200 cm of biliopancreatic limb and without measurement of the total bowel length). In addition, they did not observe significant weight loss differences between groups after 2 years of follow-up. The greater malabsorption (shorter common limb) of our OAGB allows for the obtention of greater weight loss, and it also limits the weight regain. Despite the fact that weight loss is not completely linked to T2D remission, as the improvement of the glycemic parameters after bariatric surgery appears before obtaining significant weight loss, weight regain has been related with a recurrence of comorbidities [13]. Thus, the weight loss obtained is a relevant parameter to evaluate the success of a bariatric approach, as has been shown in our OAGB group. 

In the present study, we include only patients with T2D, and obviously these subjects present more increased rates of metabolic comorbidities (hypertension and dyslipidemia) than the general morbidly obese population. Consequently, it could be expected that weight loss could be reduced in this selected population. Comparing the weight loss results obtained in the present study with data obtained in a previous study of our group [8], we observed that EBMIL was lower in T2D after SG (73.6% vs. 81.7% at 1 year, and 68.8% vs. 76.3% at 5 years) and after RYGB (81.2% vs. 77.3% at 1 year, and 72.5% vs. 77.1% at 5 years). However, these differences could not be observed after OAGB (101.8% vs. 100.4% at 1 year, and 97.6% vs. 97.9% at 5 years). These data show that the presence of metabolic comorbidities does not impair the weight loss outcomes after OAGB and further support the use of this technique on this population. However, the presence of metabolic comorbidities prevents the obtention of better ponderal results in patients undergoing SG or RYGB.

Five years after surgery, all the groups presented a good complete remission rate of T2D, globally overcoming 75%. However, significantly greater T2D remission rates could be observed after OAGB. In addition, when analyzing laboratory data, fasting glucose and HbA1c levels were significantly lower in the OAGB group in comparison with the other two groups. The pairwise analysis revealed that after OAGB lower levels of both glycemic parameters were observed, the RYGB group also showed inferior levels to SG. These results confirm the data reported in the YOMEGA trial [9], presenting lower levels of HbA1c after OAGB, but without reaching statistical differences in the complete remission rates. Notwithstanding, they showed poorer T2D complete remission rates when compared to our sample. This could be partly justified by a closer follow-up in our subjects, probably associated with closer contact with our dietitian and better adhesion to a correct postoperative diet, leading to improved glycemic control. Lee et al. [16] do not define remission of comorbidities, but they presented slightly lower fasting glucose levels after OAGB without statistical significance. Thus, only 40 patients per group were analyzed. Altogether, these results show a clear trend towards obtaining greater T2D remission rates in the OAGB group, but significant differences could not be obtained, probably because the sample size was insufficient. Further studies with an increased number of patients must corroborate this affirmation.

OAGB obtained a complete remission of DL at all the time points: 5 years after RYGB the remission rate was 69.8%, while after SG it did not reach 30%. Moreover, significantly lower levels of triglycerides and total cholesterol were obtained after OAGB when compared with the other two techniques. In the YOMEGA trial, the aim was to analyze the biological lipid profile and report no significant differences between OAGB and RYGB. However, the criteria of dyslipidemia remission and the number of patients under statin treatment were not described. Thus, the results should be interpreted with caution [9].

In our opinion, the most life-threatening nutritional complication after bariatric surgery is hypoproteinemia, as it is the most difficult deficiency to be supplemented once oral absorption is impaired. We have already reported that after OAGB with measurement of the total bowel length and customizing the limb lengths according to a percentage rather than to a fixed measure, the risk of hypoproteinemia can be reduced [7]. The YOMEGA trial could not demonstrate significantly different incidences of malnutrition after OAGB or RYGB, and the biological data did not show significant differences in albumin or prealbumin values between groups [9]. However, they described a greater incidence of ferropenic anemia and lower hemoglobin levels after 2 years of follow-up among the patients undergoing OAGB. This has been also reported by other authors [17]. Lee et al. [16] described significantly lower hemoglobin levels after OAGB compared to RYGB, and Parmar and Mahawar published a 7% rate of ferropenic anemia after OAGB [18]. In our series, this could not be confirmed, as there were no significant differences in hemoglobin levels or iron deficiencies between OAGB and RYGB. Notwithstanding, the lowest iron deficiencies between the three groups were determined after SG.

Excluding iron deficiency, there were no other significant differences in mineral and vitamin deficiencies between groups. In our previous study comparing the three techniques, significantly lower folic acid deficiencies were also observed after SG, but there were no significant differences in deficiencies or specific supplementation needs in any of the other vitamins or minerals (8). This contrasts with the results of the YOMEGA study. They describe the confusing term “nutritional complications”, defined as at least one vitamin deficiency, malnutrition, anemia, or iron deficiency, or a combination of these, and report 21% of nutritional complications after OAGB and none after RYGB [9]. The absence of deficiencies after RYGB is an outstanding result. Higa et al. [15] reported that less than 20% of the patients remain nutritionally intact during the follow-up after RYGB. Thus, the validity of these results of the YOMEGA trial must be questioned. It is true that supplementation after bariatric surgery—even more so after malabsorptive operations—is of extreme importance. However, the best supply for nutrients, even after malabsorptive procedures, is the diet. The strict postoperative follow-up by the dietitian may probably justify the low rates of nutritional deficiencies in our series, despite a moderate multivitamin and mineral supplementation.

The main limitation of this study is the retrospective evaluation. Further studies focused on the T2D population, mostly well-designed randomized clinical trials, are still necessary to confirm our results. In conclusion, OAGB obtained greater weight loss and remission of T2D and dyslipidemia than RYGB or SG. Excluding lower iron deficiencies after SG, there were no significant differences in nutritional deficiencies between groups. In our opinion, OAGB, customizing the limb lengths according to a percentage rather than to a fixed measure, is a superior technique to SG and RYGB in terms of greater weight loss and remission of comorbidities, but with similar nutritional deficiencies. For T2D patients, most of them with metabolic syndrome associated, OAGB is not only a safe alternative, but it may be considered the gold-standard approach.

## 5. Conclusions

This study describes the results of weight loss, remission of comorbidities and nutritional deficiencies of SG, RYGB and OAGB in patients with type 2 diabetes (DM2). Long-term results with greater weight loss were obtained in OAGB, as was remission of dyslipidemia with respect to the RYGB and SG techniques. A greater trend was observed in the control of DM2, with lower glucose and glycosylated hemoglobin after OAGB. Except for iron deficiencies that were lower after SG, there were no significant differences in nutritional deficiencies between the different surgical techniques analyzed.

## Figures and Tables

**Table 1:** Distribution of age, gender, preoperative anthropometric measures and distribution of comorbidities between groups.

	SG (*n* = 83)	RYGB (*n* = 152)	OAGB (*n* = 123)	*p*-Value
Age (years) (Mean ± SD)	43.5 ± 10.2	44.1 ± 11.6	42.4 ± 11	0.776
Females/Males (N)	63/20	120/32	90/33	0.532
Weight (Kg) (Mean ± SD)	117.4 ± 25.4	115.1 ± 24.1	113.6 ± 23.6	0.756
BMI (Kg/m^2^) (Mean ± SD)	45.2 ± 9.2	43.6 ±8.9	43.8 ± 9.2	0.621
T2D (%)	100%	100%	100%	1
Duration of T2D (years) (Mean ± SD)	6.3 ± 3.8	6 ± 4.1	6.6 ± 4.3	0.679
Antidiabetic oral drugs (%)	100%	100%	100%	1
Insulin therapy (N, %)	18 (21.7%)	36 (23.7%)	29 (23.6%)	0.934
Hypertension (N, %)	53 (63.9%)	95 (62.5%)	81 (65.9%)	0.847
Dyslipidemia (N, %)	49 (59%)	96 (63.2%)	76 (61.8%)	0.824

SD: standard deviation; N: number of cases; %: percentage; SG: sleeve gastrectomy; RYGB: Roux-en-Y gastric bypass; OAGB: One-Anastomosis gastric bypass; BMI: body mass index; T2D: type 2 diabetic.

**Table 2:** Postoperative BMI and excess BMI loss (EBMIL) between groups.

	SG (*n* = 83)	RYGB (*n* = 152)	OAGB (*n* = 123)	*p*-Value (ANOVA)	*p*-Value (SG Vs. RYGB)	*p*-Value (SG Vs. OAGB)	*p*-Value (OAGB Vs. RYGB)
	1 year postoperative						
BMI (Kg/m^2^)	31.3 ± 7.2	30.4 ± 6.9	24.7 ± 5.8	0.001	0.762	0.001	0.001
EBMIL (%)	73.6 ± 23.6	77.3 ± 15.3	101.8 ± 2.5	0.001	0.483	0.001	0.001
	2 years postoperative						
BMI (Kg/m^2^)	29.4 ± 5.7	28.8 ± 5.2	24.8 ± 5.3	0.001	0.713	0.001	0.001
EBMIL (%)	80.5 ± 17.8	81.9 ± 14.6	101.2 ± 2.1	0.001	0.846	0.001	0.001
	5 years postoperative						
BMI (Kg/m^2^)	32.4 ± 5.7	30.9 ± 5.9	25.9 ± 5.9	0.001	0.592	0.001	0.001
EBMIL (%)	68.8 ± 18.6	72.5 ± 16.4	97.6 ± 9.9	0.001	0.378	0.001	0.001

ANOVA: analysis of variance; BMI: body mass index; EBMIL: excess BMI loss.

**Table 3:** Remission of comorbidities between groups.

	SG (*n* = 83)	RYGB (*n* = 152)	OAGB (*n* = 123)	*p*-Value
1 year postoperative				
T2D (N, %)	68 (81.9%)	127 (83.6%)	114 (91.9%)	0.038
HT (N, %)	41 out of 53 (77.4%)	80 out of 95 (84.2%)	72 out of 81 (88.9%)	0.200
DL (N, %)	22 out of 49 (44.9%)	79 out of 96 (82.3%)	76 out of 76 (100%)	0.001
2 years postoperative				
T2D (N, %)	66 (79.5%)	127 (83.6%)	114 (91.9%)	0.018
HT (N, %)	39 out of 53 (73.6%)	80 out of 95 (84.2%)	71 out of 81 (87.6%)	0.097
DL (N, %)	19 out of 49 (38.8%)	78 out of 96 (81.2%)	76 out of 76 (100%)	0.001
5 years postoperative				
T2D (N, %)	63 (75.9%)	122 (80.3%)	110 (89.4%)	0.029
HT (N, %)	32 out of 53 (60.4%)	69 out of 95 (72.6%)	65 out of 81 (80.2%)	0.086
DL (N, %)	13 out of 49 (26.5%)	67 out of 96 (69.8%)	76 out of 76 (100%)	0.001

T2D: type 2 diabetic; HT: hypertension; DL: dyslipidemia.

**Table 4:** Remission of T2D stratified by insulin or non-insulin preoperative treatment.

Sleeve Gastrectomy	Insulin Therapy (*n* = 18)	Non-Insulin Therapy (*n* = 65)	*p*-Value
1 year postoperative	13 (72.2%)	55 (84.6%)	0.226
2 years postoperative	12 (66.7%)	54 (83.1%)	0.094
5 years postoperative	11 (66.1%)	52 (80%)	0.054
RYGB	*n* = 36	*n* = 116	
1 year postoperative	23 (63.9%)	104 (89.6%)	0.001
2 years postoperative	23 (63.9%)	104 (89.6%)	0.001
5 years postoperative	22 (61.1%)	100 (86.2%)	0.001
OAGB	*n* = 29	*n* = 94	
1 year postoperative	24 (82.7%)	90 (95.7%)	0.019
2 years postoperative	24 (82.7%)	90 (95.7%)	0.019
5 years postoperative	22 (75.9%)	88 (93.6%)	0.001

**Table 5:** Remission of T2D in patients under preoperative insulin treatment between groups.

	SG (*n* = 18)	RYGB (*n* = 36)	*p*-Value (SG vs. RYGB)	OAGB (*n* = 29)	*p*-Value (SG vs. OAGB)	*p*-Value (RYGB vs. OAGB)
1 year postoperative	13 (72.2%)	23 (63.9%)	0.54	24 (82.7%)	0.35	0.09
2 years postoperative	12 (66.7%)	23 (63.9%)	0.84	24 (82.7%)	0.21	0.09
5 years postoperative	11 (66.1%)	22 (61.1%)	1	22 (75.9%)	0.28	0.21

**Table 6:** Glycemic and lipid profile at 1, 2 and 5 years after surgery.

	SG (*n* = 83)	RYGB (*n* = 152)	OAGB (*n* = 123)	*p*-Value (ANOVA)	*p*-Value (SG Vs. RYGB)	*p*-Value (SG Vs. OAGB)	*p*-Value (OAGB Vs. RYGB)
Baseline							
Glucose (mg/dL)	107.8 ± 29.4	106.9 ± 22.3	105.5 ± 18.2	0.432			
HbA1c (%)	5.9 ± 1	5.8 ± 0.8	5.7 ± 0.9	0.381			
Triglycerides (mg/dL)	157.5 ± 71.7	156.9 ± 70.7	151 ± 76.8	0.356			
Cholesterol (mg/dL)	200.6 ± 43.5	209.3 ± 39.6	208.4 ± 38	0.180			
HDL-cholesterol (mg/dL)	47.7 ± 13	49.7 ± 8.1	49.9 ± 13.5	0.437			
1 year postoperative							
Glucose (mg/dL)	87.2 ± 19.4	83.4 ± 11.7	81.2 ± 7.6	0.006	0.324	0.002	0.468
HbA1c (%)	5.7 ± 0.8	5.4 ± 0.6	5.1 ± 0.4	0.01	0.298	0.007	0.312
Triglycerides (mg/dL)	91.4 ± 30.4	87.1 ± 33.3	80.9 ± 33.5	0.001	0.156	0.001	0.018
Cholesterol (mg/dL)	203.7 ± 38.5	166.8 ± 25.6	151.5 ± 29.2	0.001	0.007	0.001	0.09
HDL-cholesterol (mg/dL)	57.6 ± 16.5	50.6 ± 11.3	57.9 ± 14	0.016	0.034	0.344	0.008
2 years postoperative							
Glucose (mg/dL)	86.5 ± 12.5	83.5 ± 12.5	80.4 ± 7.1	0.012	0.226	0.002	0.296
HbA1c (%)	5.8 ± 0.7	5.4 ± 0.6	5.1 ± 0.7	0.018	0.212	0.004	0.331
Triglycerides (mg/dL)	91.1 ± 30.3	85.1 ± 46.1	73.1 ± 27.4	0.001	0.154	0.001	0.022
Cholesterol (mg/dL)	197.1 ± 43.1	172.9 ± 31.6	160.2 ± 32.8	0.001	0.022	0.001	0.301
HDL-cholesterol (mg/dL)	60.4 ± 15.2	57.9 ± 12.9	63.9 ± 13.3	0.032	0.046	0.397	0.017
5 years postoperative							
Glucose (mg/dL)	91.4 ± 13	85.4 ± 9.1	82.1 ± 7.9	0.015	0.285	0.004	0.315
HbA1c (%)	5.7 ± 0.4	5.5 ± 0.5	5.2 ± 0.5	0.022	0.385	0.010	0.447
Triglycerides (mg/dL)	113.6 ± 52.8	102.1 ± 30.1	88.9 ± 47.8	0.001	0.288	0.001	0.041
Cholesterol (mg/dL)	203.1 ± 31.6	175.8 ± 31.3	161.1 ± 33.3	0.001	0.014	0.001	0.277
HDL-cholesterol (mg/dL)	58.3 ± 15.9	47.4 ± 14.6	57.4 ± 15	0.001	0.001	0.401	0.001

NS: Non-significant.

**Table 7:** Hemoglobin, albumin and total protein levels at 1, 2 and 5 years after surgery.

	SG (*n* = 83)	RYGB (*n* = 152)	OAGB (*n* = 123)	*p*-Value (ANOVA)
Baseline				
Hemoglobin (g/dL)	13.9 ± 0.8	13.7 ± 0.9	13.6 ± 1	0.724
Albumin (g/ dL)	4.2 ± 0.2	4.4 ± 0.3	4.3 ± 0.3	0.735
Total proteins (g/ dL)	7.4 ± 0.5	7.3 ± 0.4	7.2 ± 0.4	0.320
1 year postoperative				
Hemoglobin (g/ dL)	13.3 ± 1.1	13.1 ± 0.9	13 ± 1.2	0.707
Albumin (g/ dL)	3.9 ± 0.5	3.8 ± 0.4	3.8 ± 0.5	0.812
Total proteins (g/ dL)	6.9 ± 0.5	6.7 ± 0.4	6.6 ± 0.4	0.164
2 years postoperative				
Hemoglobin (g/ dL)	13.3 ± 1.1	13.2 ± 1.2	13 ± 1.3	0.821
Albumin (g/ dL)	4.1 ± 0.7	3.9 ± 0.5	3.8 ± 0.5	0.621
Total proteins (g/ dL)	6.9 ± 0.4	6.7 ± 0.4	6.6 ± 0.4	0.179
5 years postoperative				
Hemoglobin (g/ dL)	13.4 ± 1.3	13.2 ± 1.4	13 ± 1.4	0.432
Albumin (g/ dL)	4.2 ± 0.7	4 ± 0.7	3.8 ± 0.6	0.286
Total proteins (g/ dL)	7.2 ± 0.4	7.1 ± 0.3	7 ± 0.3	0.285
